# Zerumbone induced apoptosis in liver cancer cells via modulation of Bax/Bcl-2 ratio

**DOI:** 10.1186/1475-2867-7-4

**Published:** 2007-04-03

**Authors:** SA Sharifah Sakinah, S Tri Handayani, LP Azimahtol Hawariah

**Affiliations:** 1 School of Bioscience and Biotechnology, Faculty of Science and Technology, National University of Malaysia (UKM), 43600 Bangi, Selangor, Malaysia

## Abstract

**Background:**

Zerumbone is a cytotoxic component isolated from *Zingiber zerumbet *Smith, a herbal plant which is also known as lempoyang. This new anticancer bioactive compound from *Z. zerumbet *was investigated for its activity and mechanism in human liver cancer cell lines.

**Results:**

Zerumbone significantly showed an antiproliferative activity upon HepG2 cells with an IC50 of 3.45 ± 0.026 μg/ml. Zerumbone was also found to inhibit the proliferation of non-malignant Chang Liver and MDBK cell lines. However the IC_50 _obtained was higher compared to the IC_50 _for HepG2 cells (> 10 μg/ml). The extent of DNA fragmentation was evaluated by the Tdt-mediated dUTP nick end labelling assay which showed that, zerumbone significantly increased apoptosis in HepG2 cells in a time-course manner. In detail, the apoptotic process triggered by zerumbone involved the up-regulation of pro-apoptotic Bax protein and the suppression of anti-apoptotic Bcl-2 protein expression. The changes that occurred in the levels of this antagonistic proteins Bax/Bcl-2, was independent of p53 since zerumbone did not affect the levels of p53 although this protein exists in a functional form. Western blotting analysis for Bax protein was further confirmed qualitatively with an immunoassay that showed the distribution of Bax protein in zerumbone-treated cells.

**Conclusion:**

Therefore, zerumbone was found to induce the apoptotic process in HepG2 cells through the up and down regulation of Bax/Bcl-2 protein independently of functional p53 activity.

## Background

Carcinogenesis is composed of a multi-stage process of initiation, promotion and progression. In the steady-state, cell division must be counterbalanced by cell death. This important active process of cell death is known as apoptosis or programmed cell death. The term "apoptosis" was introduced by Kerr to describe a form of hepatocellular cell death in ischaemic liver disease [1]. Apoptosis has been recognized as a tightly controlled mechanism involving death factors and death receptors in the control of cell proliferation. The recognition of tumor development involves an imbalance between cell proliferation and apoptotic cell death, which is the current dogma in tumor biology [2]. Evidence showed that hepatocellular apoptosis is essential in all three stages of hepatogenesis, involving the initial genotoxic insult (initiation), through the clonal expansion from a premalignant to a tumorous lesion (promotion) and finally to the progression of tumor growth by further clonal expansion [3].

Hepatocellular carcinoma (HCC) derived from hepatocytes is one of the most common malignancies throughout the world. It is characterized by its high incidence in hepatitis B virus-associated cirrhotic liver disease [4] and other risk factors such as hepatitis C virus, aflatoxin, sex, hormones and some metabolic diseases [4,5]. The different epidemiology distributions of HCC have facilitated the identification of these associated risk factors series [4]. Thus, a great deal of research has been turned towards novel chemotherapeutic drugs from the plant kingdom in search of cancer inhibitors and cures. Pezzuto reported that the bioactive components obtained from herbal plants have high potential in preventing and controlling carcinogenesis [6].

Zingiberaceae is one of the largest families of the plant kingdom most frequently used as raw material for making various traditional medicine formulations that are commonly sold in the market [7,8]. It is an important natural resource that provides many useful products for food, spices, medicines, dyes perfume and aesthetics to man. Traditionally, the rhizome of *Zingiber zerumbet *are employed as medicine in relieving stomachache, macerated in alcohol which is regarded as tonic and depurative. Besides, it is also used as the spice ginger and a novel food factor for mitigating experimental ulcerative colitis.

Scientific research towards *Zingiber zerumbet *proved that it contained a suppressive effect which was conducted by a bioactive compound, zerumbone. It is also has been found able to exert antitumor activity [9,10], anti-inflammatory effects and possesses antiproliferative potentials in a variety of cell culture [11]. It is identified that the inhibition of Epstein-Barr virus (EBV) early antigen (EA) activation which was induced by tumor-promoters *in vitro *correlated well with the zerumbone anti-tumor promoting effect *in vivo *[10,12]. Mechanisms of inducing apoptosis in the hepatocarcinoma cells by zerumbone was carried out in vitro using a well-differentiated transformed cell line, HepG2 cells which have been widely used and considered to be a good model for liver cancer research [13].

Zerumbone is a crystalline sesquiterpene derived from the wild ginger, *Z. zerumbet*. This bioactive component has its unique structure, with a cross-conjugated ketone in an 11-membered ring, as well as an interesting biological activity [14]. Antiproliferative activity of *Z. zerumbet *is mainly modulated by the zerumbone component which is the main cytotoxic compound that constitute about 37% of the whole *Z. zerumbet *content [15]. As Murakami *et al*. stated, zerumbone also displayed a selective cytotoxic characteristic towards cancer cell lines and normal cell lines. Zerumbone was also found to inhibit the proliferation of human colonic adenocarcinoma cell lines in a dose dependent manner while less effective towards the growth of normal human dermal and colon fibroblasts [11]. Thus, this study aims to elucidate if the cytotoxic and antiproliferative action of zerumbone is mediated by the apoptotic mode of cell death.

## Results

### Effect of zerumbone on cell viability

**Figure 1:**
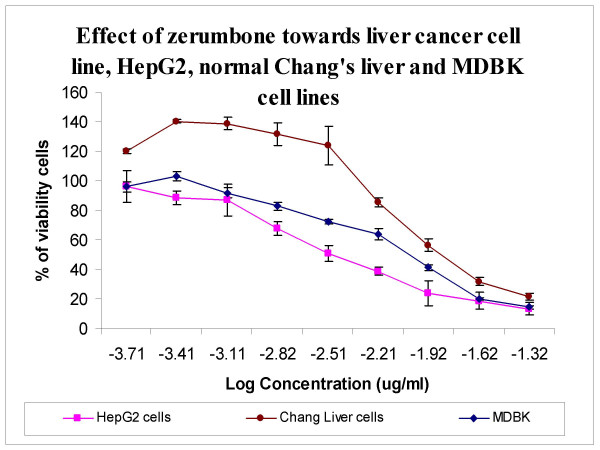
Effects of zerumbone on cell viability of HepG2 cancer cell lines and non-cancer Chang Liver and MDBK cells. Treatment of zerumbone on HepG2 cell lines significantly reduced the number of viable cells with IC_50 _values being obtained less than 5 μg/ml. Non-malignant Chang Liver cells was also affected but the IC_50 _was the highest compared to other malignant cell lines while the IC_50 _of zerumbone-treated non-malignant MDBK cells was 10.02 ± 0.03 μg/ml.

Figure 1 shows that, zerumbone was able to exert the antiproliferative effects towards human cancer cell line, HepG2 tested in time-dependent manner. The IC_50 _values which is the concentration required for 50% growth inhibition of zerumbone towards HepG2 cell viability is 3.45 ± 0.026 μg/ml. Zerumbone also inhibits the proliferation of non-malignant Chang Liver and MDBK cells with an IC_50 _value of 10.96 ± 0.059 μg/ml and 10.02 ± 0.03 μg/ml respectively whereby the IC_50 _value exceeding > 10 μg/ml is the highest compared to other cancer cells.

**Figure 2:**
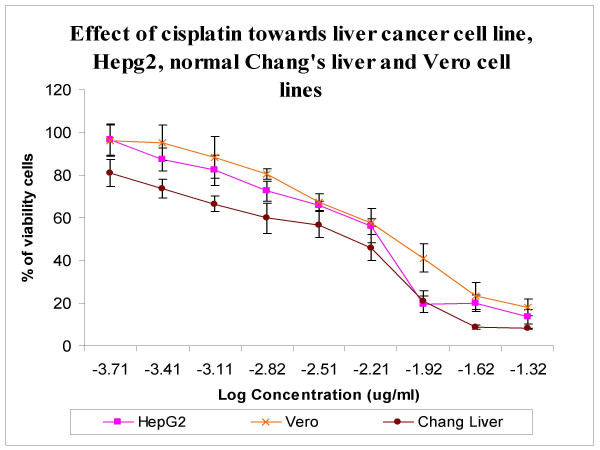
Effects of cisplatin on cell viability of HepG2 cancer cells and non-malignant Vero and Chang liver cell lines. The effectiveness of cisplatin on HepG2 cells and non-malignant Chang Liver cells did not significantly differ since the IC_50 _obtained for both malignant and non-malignant were 7 μg/ml. The IC_50 _of cisplatin-treated non-malignant Vero cells was 9.06 ± 0.044 μg/ml.

Comparatively, cisplatin, a drug with antineoplastic activity was used in this study. Cisplatin is used widely in the treatment of ovarian, bladder and testicular cancer. Cisplatin imposed an inhibitory effect on HepG2 cells with an IC_50 _value of 7.23 ± 0.036 μg/ml. Cisplatin was also found to be effective toward non malignant cells of Vero and Chang Liver with IC_50 _values of 9.06 ± 0.044 μg/ml and 7.08 ± 0.073 μg/ml respectively (Figure 2)

### Zerumbone induced apoptotic cell death

**Figure 3:**
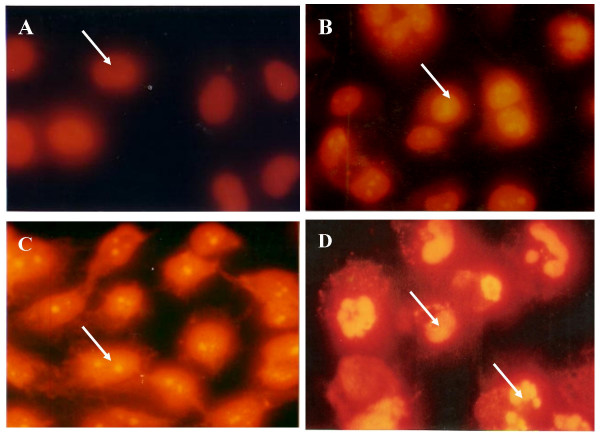
HepG2 cells were treated with 3.45 μg/ml zerumbone for 24 (B), 48 (C) and 72 (D) hours. DMSO treated HepG2 cells served as negative control (A) and thus gave TUNEL-negative results indicating less apoptotic signal. Arrows indicated cells with fragmented DNA due to apoptosis which occurred actively at the beginning of the treatment and the presence of apoptotic bodies after 72 hours at the end of treatment. Magnification: 1000×.

**Figure 4:**
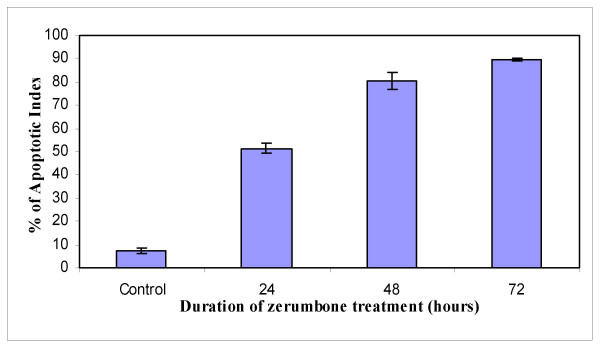
Percentages of HepG2 cell death via apoptosis after zerumbone treatment. HepG2 cell death via apoptosis increased significantly in a time-dependent manner.

**Figure 5:**
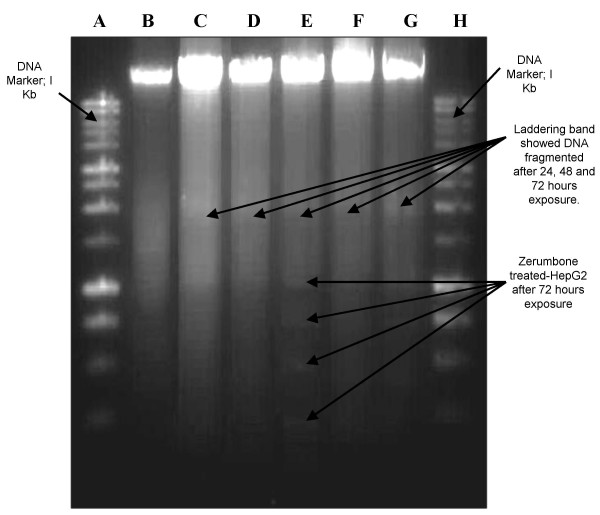
Agarose gel of electrophoresis of DNA from HepG2 cells treated with zerumbone for 24, 48 and 72 hours (Lane C, D, E). DNA fragmentations with a ladder pattern are characteristic of apoptosis. Lane A and H were the molecular marker while lane B was the negative control (untreated cells). Lane F and G showed apoptotic DNA in HepG2 cells treated with cisplatin at 24 and 72 hours.

To further define the mechanism of antiproliferative effect of zerumbone, HepG2 cells were treated with zerumbone at 3.45 μg/ml in a time-course manner to determine whether this bioactive compound induced HepG2 cell death via apoptosis or necrosis (Figure 3). Cisplatin was used as positive control while negative control was treated with DMSO (Figure 3[A]). HepG2 cells which were treated with zerumbone for 24 hours (Figure 3[B]), showed active apoptosis and the fragmented DNA were labeled with fluorescence 12 dUTP in the nuclei. At the beginning of the treatment, the intensity of the yellow fluorescence was dim. However, more fluorescence TdT-binding occurred at 48 hours of treatment, thereby indicating more cells were undergoing apoptosis (~80%) (Figure 3[C]). At 72 hours of treatment, HepG2 cells showed membrane blebbing and the presence of apoptotic bodies (Figure 3[D]). Apoptosis was also shown by the typical oligonucleosomal ladders which indicated DNA from treated cells was fragmented into 180 until 200 base pair of nucleosomal multimers (Figure 5). The same phenomenon occurred when HepG2 cells were treated with cisplatin, however the intensity of yellow fluorescence was not as bright as the fluorescence in zerumbone-treated HepG2 cells (data not shown). In HepG2 cells treated with DMSO as negative control, no fluorescence was detected in the nuclei, due to the absence of fragmented DNA (Figure 3[A]). The percentages of apoptotic cells after zerumbone treatment increased in a time-course manner with > 50% at 24 hours and ~80% by 48 hours. Untreated cells which represent the control showed only 6% of cell death via apoptosis (Figure 4 [Control]).

### Zerumbone up-regulated Bax and suppressed the expression of Bcl-2 protein

**Figure 6:**
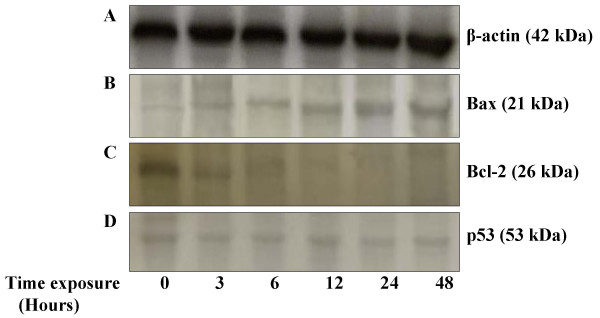
*In vitro *expression of Bax and Bcl-2 protein for 0, 3, 6, 12, 24 and 48 hours. p53 expression did not change significantly since the p53 was constitutively expressed in both control and treated cells.

**Figure 7:**
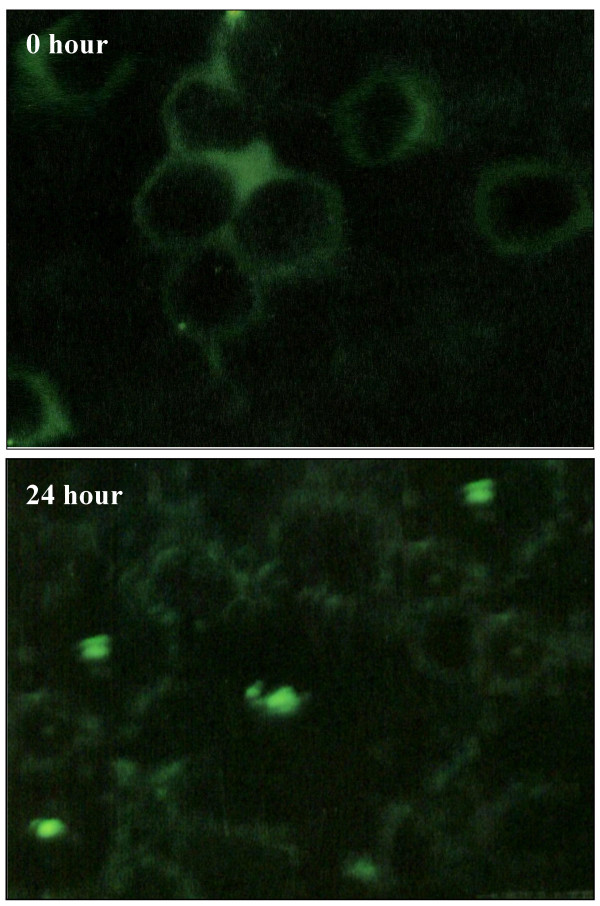
Immunostaining of Bax protein showed a low level of Bax in the untreated HepG2 cells. However, the immunofluorescence of Bax protein increase and can be seen after 24-h treatment with zerumbone.

To determine which apoptosis-related proteins are regulated by zerumbone, the expression of p53, Bax and Bcl-2 protein were measured after 3.45 μg/ml zerumbone treatment for 0, 3, 6, 12 and 48 hours in HepG2 cells using Western Blotting analysis (Figure 6). Exposure of HepG2 cells to zerumbone increased the pro-apoptotic protein, Bax and decreased the expression of anti-apoptotic, Bcl-2 protein. The up-regulation of Bax by zerumbone was confirmed via immunostaining (Figure 7). However, the expression of protein suppressor tumor, p53 did not show any significant changes compared to control throughout the treatment. The result implies that apoptosis induced by zerumbone may be mediated by the Bax and Bcl-2 pathways in liver cancer cells, HepG2.

## Discussion

The aim of this study was thus to elucidate the mechanism of the apoptotic effect induced by zerumbon in HepG2 cells. In Asia, medicinal herbs are used as treatment for various ailments including malignancies [16]. Previous study showed that this active compound able to exhibit versatile biological activities such as repressed insulin-like grow factor-1 and induced Waf-1 gene expression [17], glutathione S-transferase activity [18] and heat shock protein [19]. Zerumbone was also found to exert antiproliferative activity which inhibits tumor cell growth [20], induction of differentiation [21], apoptosis [22] and cytoprotective activity [23]. According to Matthes *et al*., *Z. zerumbet *from the family of Zingiberaceae has cytotoxic effects on many types of cancer cells [20] and dramatically suppresses the EBV activation [11]. The data obtained in this study revealed the inhibitory effect of zerumbone on HepG2 cancer cell growth (IC_50 _of 3.45 ± 0.026 μg/ml). Zerumbone did show the ability to act as a cytoselective anticancer agent since zerumbone was three times less effective towards non-malignant Chang Liver cells (IC_50 _> 10 μg/ml) and non-malignant MDBK cells (IC_50 _= 10.02 ± 0.03 μg/ml). Previous research showed the minimum effect of zerumbone towards non-malignant MDBK cells with IC_50 _value of 7.20 ± 0.32 μg/ml in comparison with MCF-7 cells (IC_50 _= 2.49 ± 0.13 μg/ml) [24]. Hoffmann *et al*. indicated an appropriate dose of zerumbone induced a high intracellular redox potential which stopped the proliferation of cancer cells but not the normal cells [25]. This was also proven by Murakami *et al*. who reported that zerumbone inhibited the proliferation of human colonic adenocarcinoma cell line in a dose dependent manner while the growth of normal human dermal (2F0-C25) was less affected [11]. Thus, the effects of zerumbone were specific towards tumor cells.

However the effect of cisplatin was not cytoselective since its antiproliferative effect was towards both cancerous and non-cancerous cells. Recent evidence indicated that the nephrotoxic effects of cisplatin is still a common adverse effect in both adults and children even with the use of hyperhydration and other protective measures [26-28]. Our data indicated that cisplatin gave a low IC_50 _value towards normal Chang Liver cells (IC_50 _7.08 ± 0.073 μg/ml) and normal Vero cells (IC_50 _9.06 ± 0.044 μg/ml). However, the effect of cisplatin towards cancerous cells especially on HepG2 cells show that it is not as effective as zerumbone. This is because, the effects of zerumbone towards HepG2 cells was (IC_50 _= 3.45 ± 0.026 μg/ml) twice lower in comparison with the effects of cisplatin (IC_50 _7.08 ± 0.073 μg/ml). Thus, these finding suggested that zerumbone has more ability to inhibit the proliferation of human liver cancer, HepG2 cells compared to cisplatin.

To confirm that zerumbone-treated cell death was via apoptosis, the extent of DNA fragmentation was analyzed and Apoptotic Index calculated. Apoptotic Index (AI) is described as percentage of apoptotic cells and apoptotic bodies within the overall population of total cells [29]. When HepG2 cells were treated with zerumbone (3.45 μg/ml), TUNEL-positive cells detected at 24 hours of treatment was > 50%. Gavrielli *et al*. reported that in the early process of apoptosis, DNA fragmentation occurs at the periphery of the nucleus within minutes [30] while lysosomal degradation ended within hours depending on cell type and tissue [31-33]. This can be seen in the increase of apoptotic scores ~80% by 48 hours and 90% after 72 hours of zerumbone treatment. Morphologically, in the late stage of cell death, the effect of zerumbone produced fragmentation of condensed chromatin into several discrete mass. Untreated control cells only recorded ~6% of apoptotic cells. Figure 3(B), 3(C) and 3(D) showed HepG2 cells underwent DNA fragmentation with similar characteristics of apoptotic cells [34-36].

Zerumbone was also found able to cleave the double-stranded DNA into fragments of 180–200 base pair. This can be observed after the treatment with zerumbone at concentration of 3.45 μg/ml. DNA fragmentation is the primary physiological characteristic which indicate an early event in apoptosis and it represents a point of no return from the path to cell death. This is due to no more new cellular protein will be synthesized for cell survival. As shown in Figure 5, multiple-unit of apoptotic DNA ladder was detected in zerumbone treated HepG2 cells and the apoptotic signal increased with the duration of treatment. Previous study showed that, the cleavage of double-stranded DNA in apoptotic DNA degradation occurs via the activation of endogenus Ca^2+^/Mg^2+ ^dependent endonuclease that specifically cleaves between nucleosomes to produce DNA fragments that are multiples of ~180 base pair [37,38].

In further analysis, we demonstrated that zerumbone markedly inhibited the variability of HepG2 cells and this was a consequence of the induction of apoptosis as evidenced by Western Blot profiles, TUNEL assay and DNA fragmentation analysis. The data showed that the fundamental event that occurred when HepG2 cells were treated with zerumbone, is a marked decrease in the level of these two antiapoptotic and proapoptotic factors. The susceptibility of tumor cells to the induction of apoptosis by chemotherapeutic agents is controlled by the ratio of Bcl-2/Bax proteins in the mitochondria [39]. The pro-apoptotic activity of Bax and the related protein was held at bay by the formation of complexes with anti-apoptotic protein, Bcl-2. When cells in culture received death signals, Bax moves to mitochondria and other membrane sites and triggers a catastrophic transformation of mitochondrial function which includes release of cytochrome *c *to the surrounding cytosol, loss of transmembrane potential and induction of mitochondrial permeability transition events that result in apoptotic cells [40]. From data obtained (Figure 6), treatment of liver cancer cells elicit the down-regulation in Bcl-2 and significantly up-regulated the expression of Bax. Takada *et al*. reported, the downregulation of Bcl-2 protein led to the potentiation of apoptosis induced by cytokines and chemotherapeutic agents [41]. In particular, an important role seems to be exerted by Bcl-2 when present at a higher level in untreated HepG2 cells. Bcl-2 reacts on interceding and blocking the Bax induced events at several levels including preventing the Bax redistribution after a death signal [42] Therefore zerumbone act in balancing the ratio of Bax/Bcl-2 and the increase of Bax protein in HepG2 cells seems to contribute to the apoptotic effect.

In contrast to the expression of p53 protein, the activation of apoptosis by zerumbone is independent of p53 since the expression levels of p53 did not show any significant increase after zerumbone treatment (Figure 6). However, Muller *et al*. reported p53 gene in HepG2 cells was not mutated and existed as a functional wild form [43]. Thus, this showed that zerumbone can induce apoptosis of HepG2 cells in p53 deficiency. Although p53 is a transcription factor that involves stabilization of the protein and establishes programmes for apoptosis, senescence, and repair in response to a variety of cellular stresses, including DNA damage, hypoxia, nutrient deprivation and untimely expression of oncogenes [44-46] the relative importance of p53-independent and p53-dependent apoptotic mechanisms in suppressing tumorigenesis remains unclear. A lot of anticancer drugs such as methotrexate, bleomisin [43] cause the death of cancer cells via activation of p53 tumor suppressor gene. However this strategy does not work since many types of cancer arose by spontaneous occurrence of mutation in p53 gene or inactivation of p53 protein function by viral protein, such as Hepatitis B virus × [47].

## Conclusion

Our study demonstrates that zerumbone induced apoptosis in HepG2 cells by inhibiting the proliferation of cancer cells. The inhibition was caused by decreasing the levels of anti-apoptotic protein, Bcl-2 and up-regulation of proapoptotic Bax without involving p53. Therefore, we suggest that zerumbone could be further investigated as a new alternative chemotherapeutic agent for human hepatoma.

## Materials and methods

### Chemicals

Dulbecco's modified Eagle's medium (DMEM), dimethyl sulfoxide (DMSO), penicillin, propidium iodide, streptomycin, fungizon, miramycin and tryspin-EDTA were bought from Sigma Chemical Co. (St. Louis, MO, USA). Fetal bovine serum (FBS) was obtained from GIBCO BRL (Gaithersburg, MD). TUNEL Kit was purchased from Promega (Madison, WI). All other chemicals used were of the highest pure grade available. Cell culture plasticware were from Nunc Co. (Denmark).

### Cell Culture

Human liver cancer cells (HepG2), non-malignant cells of Chang's Liver, MDBK and Vero were obtained from American Type Cell Culture Collection (ATCC), Maryland, USA. All cultured cells were maintained in the logarithmic phase of growth in DMEM supplemented with 10% fetal bovine serum (GIBCO BRL), penicillin-streptomycin, fungizon and miramycin at 37°C in a humidified incubator with 5% CO_2 _and 95% air. Cultures were regularly examined using inverted microscope.

### Antiproliferative assay

Trypsinized cells were counted using hemocytometer and plated in a microtiter plate of 96 wells. After an overnight incubation to allow cells attachment, medium were changed and 0.2 ml of new supplemented medium were added in each well. Cells were then treated with 2 μl zerumbone in a dose dependent-manner, 0.1% DMSO (negative control) and cisplatin (positive control) and were incubated at 37°C, 5% CO_2 _for 72 hours. Each concentration of the compounds was assayed in triplicates. The antiproliferative effect of zerumbone was monitored employing the Methylene Blue method [48]. The absorbance was measured on an ELISA reader at a test wavelength of 660 nm.

### TUNEL assay

The mode of cell death induced by zerumbone was determined by morphological observations done with TUNEL assay. Cells were grown on microscope slides and were treated with zerumbone based on the IC_50 _value obtained from the antiproliferative assay. The fragmented DNA of apoptotic cells was quantified by Tdt-mediated dUTP nick end labelling (TUNEL) with the Apoptotic Detection Kit (Promega Inc. USA). Briefly, the cells were fixed with 4% methanol-free paraformaldehyde at 4°C and washed with phosphate-buffered saline (PBS) for 30 minutes. Each slide was then added with 0.1 ml of equilibrium buffer and covered with parafilm for 10 minutes at 37°C. A mixture of 1 μl TdT (terminal deoxynucleotidil transferase) enzyme, 5 μl nucleotide mix and 45 μl equilibrium buffers were prepared in the dark and 50 μl of the mixture were added on each slides. Next, the slides were incubated for 1 or 2 hours at 37°C in a container, to protect it from any light source. After that 2× SSC were added for 15 minutes in a room temperature to stop the TdT enzyme's reaction. After washing with PBS which is to eliminate the unbound fluorescen-12-dUTP, the slides were immersed in propidium iodide for 15 minutes in the dark to stain the cells. Slides were dried after rinsing with deionized water and cover slip was later overlaid on the cell area of the slides. This assay specifically detects apoptotic cells when examined through the Zeiss fluorescent microscope.

### DNA fragmentation

Soluble DNA from the cells was extracted by a previously reported method. briefly, after washing with PBS, cells were lysed with 500 μl of lysis buffer (10 mM Tris-HCl, pH 7.8; 5 mM EDTA and 0.5% sodium dodecyl sulfat) containing 50 μg/ml proteinase K and incubated at 45°C for 3 hour. The resulting products were extracted twice with phenol: chloroform: isoamyl alcohol 25:24:1, and chloroform once and then treated with 100 μg/ml of RNase A for an hour at 37°C. DNA was extracted again with chloroform twice to ensure a complete removal of phenol. DNA was precipitated with 70% ethanol (dissolved in Tris-EDTA buffer) and analyzed by 1.5% agarose gel electrophoresis.

### Western blotting

Protein expression of Bax, Bcl-2 and p53 were analyzed by Western Blotting. Cells were harvested, aliquoted and lysed in lysis-buffer. Protein sample (30 ug) from both zerumbone-treated and untreated cells were separated on 15% SDS-polyacrylamide gels. After electrophoresis, the proteins were blotted onto polyvinyl-difluoride (PVDF) membranes (PolyScreen, NEN Life Sciences, USA). The membranes were dried, preblocked with 5% non-fat milk in PBS-Tween (0.1%), then incubated with the primary antibodies (p53, Bax and Bcl-2) diluted in 1: 2000. The p53 antibody used can detect both wild-type and mutant p53 protein. The secondary antibody used was horseradish peroxidase labeled to rabbit or mouse IgG. A densitometry analysis was performed using a GS 670 Imaging Densitometer with software Molecular Analyst (BioRad, Hercules, USA) after exposure on a Kodak OMAT X-ray film. The membranes were reprobed with β-actin antibodies (Sigma) as an internal control and to confirm equal loading.

## List of abbreviations

ATCC, American Type Cell Culture Collection; Bax, Bcl-2-associated × protein; Bcl-2, B-cell lymphoma-2; Ca^2+^, calcium ion; Chang liver cells, normal liver cells; CO_2_, carbon dioxide; DMEM, Dulbecco's modified Eagle's medium; DMSO, dimethylsulfoxide; DNA, deoxyribonucleic acid; dUTP, deoxyuridine triphosphate; EBV, Epstein-Barr virus; EDTA,; ELISA, Enzyme Linked Immuno Sorbent Assay; FBS, fetal bovine serum; HCC, hepatocellular carcinoma; HCl, hydrochloride acid; HepG2 cells, liver cancer cells; IC_50_, inhibition concentration to kill 50% of cells population; IgG, ; MDBK cells, Madin Darby Bovine Kidney cells; Mg^2+^, magnesium ion; PBS, phosphate-buffered saline; PVDF, polyvinyl-difluoride; SDS, sodium dodesil sulphate; SSC, ; TdT, Terminal Deoxynucleotidyl Transferase; TUNEL, Tdt-mediated dUTP nick-end labeling; Vero cells, ; Waf-1, ; 2F0-C25 cells, normal human dermal cells

## Competing interests

Before this, there are other researchers working on this active compound, zerumbone. However, they are working towards other cancer cells but not liver cancer cell. Furthermore, the research has been conducted for 3 years ago.

## Authors' contributions

SS carried out the whole research and drafted the manuscript; TH carried out research on the antiproliferation assay using cisplatin; AHLP conceived of the study, and participated in its design and coordination and helped to draft the manuscript. All authors read and approved the final manuscript.
